# Luminescent chemosensors by using cyclometalated iridium(iii) complexes and their applications

**DOI:** 10.1039/c6sc04175b

**Published:** 2016-11-02

**Authors:** Dik-Lung Ma, Sheng Lin, Wanhe Wang, Chao Yang, Chung-Hang Leung

**Affiliations:** a Department of Chemistry , Hong Kong Baptist University , Kowloon Tong , Hong Kong , China . Email: edmondma@hkbu.edu.hk; b State Key Laboratory of Quality Research in Chinese Medicine , Institute of Chinese Medical Sciences , University of Macau , Macao , China . Email: duncanleung@umac.mo

## Abstract

Luminescent metal complexes have found increasing use in multiple areas of science and technology, including in chemosensing, light-emitting devices and photochemistry. In particular, the use of cyclometalated iridium(iii) complexes as chemosensors has received increasing attention in the recent literature. Phosphorescent metal complexes enjoy a number of advantages (*e.g.*, long-lived phosphorescence, high quantum efficiency and modular syntheses) that render them as suitable alternatives to organic dyes for sensing a variety of analytes. This review describes recent examples of cyclometalated iridium(iii) complexes that act as luminescent chemosensors for cations, anions or small molecules.

## Abstract 2

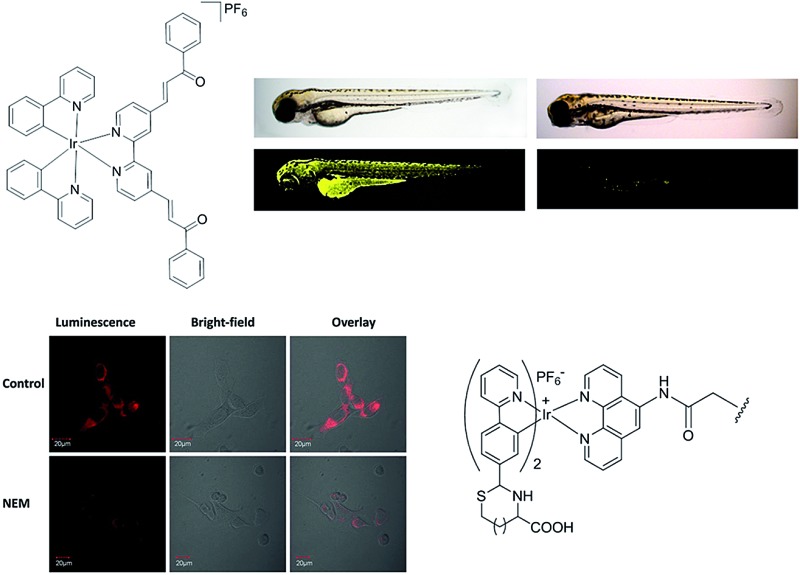
This review describes recent examples of cyclometalated iridium(iii) metal complexes that act as luminescent chemosensors for cations, anions or small molecules.

## 1 Introduction

Over the last few decades, luminescent metal complexes have received expanding interest from both academia and industry in a number of fields, including optoelectronics, photochemistry, and luminescent chemosensors.^1–5^ Transition-metal complexes in the second- and third-row of the d-block typically display triplet emission, which is a result of spin–orbit coupling leading to singlet–triplet mixing due to the heavy atom effect. In contrast, organic dyes are usually singlet emitters and emit *via* a short-lived fluorescence modality. Transition-metal complexes also differ from organic fluorophores in that they can display a range of excited states, such as metal-to-ligand charge-transfer (MLCT), ligand-to-ligand charge transfer (LLCT), intraligand charge-transfer (ILCT), ligand-to-metal charge transfer (LMCT), metal–metal-to-ligand charge-transfer (MMLCT), ligand-to-metal–metal charge transfer (LMMCT) and metal-to-ligand–ligand charge-transfer (MLLCT) states.^6,7^

Transition-metal complexes offer distinct advantages that render them as suitable alternatives to organic molecules as luminescent chemosensors.^8–13^ For example, their long-lived phosphorescence can allow them to be distinguished from an autofluorescent background, which is common in biological milieu, by the use of time-resolved emission spectroscopy (TRES). This method involves setting the time gate on the spectrophotometer to be greater than the fluorescence decay time of endogenous fluorophores, such that only long-lived phosphorescent signals are recorded. This method can also be extended to luminescence imaging. Williams and co-workers have recently described how the phosphorescence of metal complexes can be exploited in phosphorescence lifetime imaging microscopy and time-resolved emission imaging microscopy.^14^

Further advantages of luminescent metal complexes include their high luminescence quantum efficiency, as well as the sensitivity of their phosphorescent behavior to changes in the local environment. This allows many metal complexes to act as chemosensors for a variety of analytes.^15–20^ Additionally, the large Stokes shift can allow excitation and emission wavelengths to be effectively separated, while the modular nature of inorganic synthesis allows facile modification of metal complexes for optimization of their luminescent or structural properties. This is because the excited states of transition-metal complexes are highly sensitive to both the nature of the metal center as well as the character of the auxiliary ligands.

Early studies on luminescent metal complexes were concentrated mainly on ruthenium(ii) and platinum(ii) complexes, including the well-known luminescent “light switch” complex [Ru(phen)(dppz)]^2+^ (where phen = 1,10-phenanthroline and dppz = dipyrido[3,2-*a*:2′,3′-*c*]phenazine) pioneered by Barton and co-workers.^21^ However, group 9 metal complexes, particularly cyclometalated iridium(iii) complexes, have attracted more recent interest as luminescent chemosensors due to their beneficial features. Compared to d^8^ square-planar platinum(ii) complexes, the octahedral structure of iridium(iii) complexes can provide an alternative scaffold for analyte recognition. Meanwhile, the iridium(iii) center can also exhibit longer lifetimes and higher quantum yields compared to ruthenium(ii). Moreover, the emission wavelengths of iridium(iii) complexes (from green to red) can be readily tuned by varying the nature of the auxiliary co-ligands.

In transition-metal complex-based chemosensors, the metal center usually functions as the signaling unit, so that the presence of the analyte can be transduced into a luminescent response. Then, the auxiliary ligands of the complex will contain a recognition unit that selectively interacts with the target analyte. The recognition unit can be attached to the metal center either through a σ-linker or a π-linker. The interaction of the analyte with the recognition unit thus changes the photophysical properties of the metal complex, allowing the complex to act as a “switch-on” or “switch-off” chemosensor for the target analyte. Additionally, recognition units that are conjugated to the metal center through a π-linker can change the emission wavelength of the metal complex when the analyte binds. This affords the possibility of ratiometric detection, which can increase accuracy particularly under conditions of high background noise.

Several review articles about chemosensors have been published, however, only a few of these have summarized metal complex-based chemosensors.^3,7,22–25^ Some of those have focused on the use of a specific motif,^23,24^ or considered only a limited range of analytes,^22,25^ while some of them have been published for several years so that new examples has not been included.^3,7^ In light of the recent expansion in the literature on cyclometalated iridium(iii) chemosensors, this review seeks to highlight recent examples of the use of cyclometalated iridium(iii) complexes as chemosensors for anions, cations and small molecules. For brevity, we focus only on recent examples that have been reported in the past five years (2012–2016). Additionally, oligonucleotide-based detection platforms are not included in this summary, as this topic has been recently covered in reviews by our group and others.^7,26,27^

## 2 Cyclometalated iridium(iii) complexes as chemosensors for the detection of cations

**Fig. 1:**
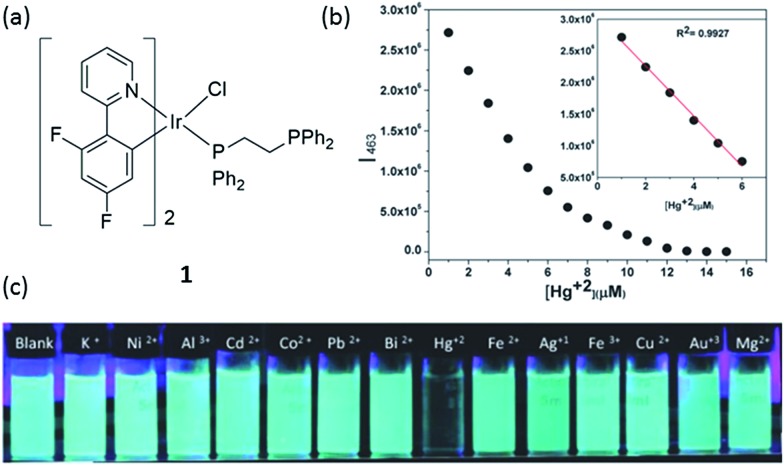
(a) Chemical structure of complex **1**. (b) The plot of the normalized emission intensity of complex **1** (10 μM) at 463 nm *vs.* Hg^2+^ concentration in dimethylformamide (DMF)–water (3 : 7) mixtures. (c) Photograph images of **1** (10 μM) upon the addition of 4 equivalents of different metal ions under UV irradiation.^29^ Reprinted figures with permission from Copyright (2014) Royal Society of Chemistry.

Mercury and its derivatives cause serious environmental and health problems. Mercury(ii) ion is usually the most common ionic form of mercury, and it may accumulate in the organs of humans or animals *via* the food chain.^28^ Laskar and co-workers developed a blue-emitting bis-cyclometalated iridium(iii) complex **1** (Fig. 1a), which showed higher sensitivity for Hg^2+^ ion than other kinds of metal ions.^29^ The phenyl-substituted diphosphines are used as ancillary ligands, with only one phosphorus atom coordinated to the chromophoric iridium(iii) center, while the other phosphorus unit remains non-coordinated and can easily interact with Hg^2+^ ions. The effect of Hg^2+^ on complex **1** was studied by UV-vis spectroscopy, which revealed a gradual shift of the band at 372 nm assigned to the spin-allowed metal-to-ligand charge-transfer (^1^MLCT) (dπ(Ir) → π*(C^N)) transition to 356 nm with two isosbestic points at 322 and 360 nm, indicating the strong interaction between **1** and the Hg^2+^ ions.

The emission titration profile of complex **1** (10 μM) with Hg^2+^ suggested that down to 170 nM of Hg^2+^ could be determined *via* a switch-off mode of detection (Fig. 1b). The selectivity of complex **1** towards Hg^2+^ over 4 equivalents of other metal ions was also demonstrated (Fig. 1c). The selectivity of **1** is believed to arise through the selective interaction of the free lone pair on the non-coordinated phosphorus atom with Hg^2+^. It should be noted that while Ag^+^ ions could induce a small decrease in emission intensity, which might be understood on the basis that d^10^ ions do not usually introduce low-energy charge-separated or metal-centered excited states to the fluorophore thus restricting electron-transfer or energy-transfer processes,^30^ the degree of intensity reducing from 1 equivalent of Hg^2+^ ions was still about 10-fold higher than that for 4 equivalents of Ag^+^ ions. Moreover, the sensitivity of complex **1** was superior to that displayed by a sulfur-containing iridium(iii) chemosensor which had a detection limit of 1.04 μM,^31^ while a sulfur-free iridium(iii) complex (pbi)_2_Ir(mtpy) with 1,2-diphenyl-1*H*-benzoimidazole (Hpbi) and 2-(5-methyl-2*H*-1,2,4-triazol-3-yl)pyridine (Hmtpy) ligands showed a detection limit of 0.25 μM for Hg^2+^.^32^

**Fig. 2:**
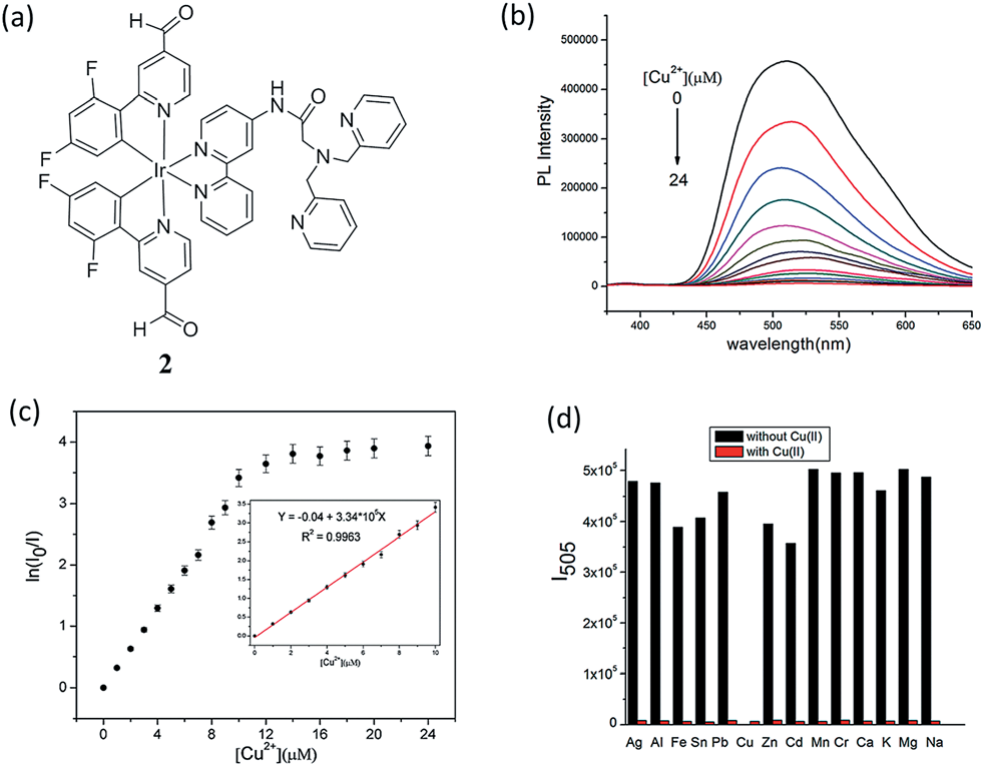
(a) Chemical structure of complex **2**. (b) The emission spectrum of **2** (10 μM) in aqueous solution with different concentration of Cu^2+^ (*λ*_ex_ = 343 nm). (c) The plot of ln(*I*_0_/*I*) *vs.* the concentration of Cu^2+^ (**2**); inset, the linear relationship of ln(*I*_0_/*I*) *vs.* the concentration of Cu^2+^ from 0 to 10 μM. (d) Emission intensity of **2** + M^*n*+^ (metal ions) at 505 nm. Black bar means introducing other metal ions (40 mM) into the blank solution and red bar means adding 2 equiv. Cu^2+^ continuously to the above mentioned solutions (*λ*_ex_ = 343 nm).^36^ Reprinted figures with permission from Copyright (2016) Royal Society of Chemistry.

The copper ion (Cu^2+^) is an essential component for a range of biological activities. Both excess and insufficient Cu^2+^ ion can lead to diseases, including Alzheimer's, Menkes, Wilson's and prion diseases.^33,34^ Furthermore, Cu^2+^ imbalance is also harmful to flora and fauna. Thus, Cu^2+^ is considered to be an environmental pollutant.^35^ Sun and co-workers reported an iridium(iii) complex functionalized with the ion-recognition unit DPA (bis(pyridin-2-ylmethyl)amine) (complex **2**, Fig. 2a) for the switch-off detection of Cu^2+^, while an amide group was utilized as a linker between the ligand 2,2′-bipyridine and DPA.^36^ DPA is a derivative of *N*,*N*,*N*′,*N*′-tetrakis(2-pyridylmethyl)ethylenediamine,^37^ and is a classical Zn^2+^ chelator with high selectivity for Zn^2+^ over alkali and alkaline-earth metal ions.^38^ While DPA has been frequently used to construct Zn^2+^ chemosensors, those sensors were often quenched by Cu^2+^ even after complexation with Zn^2+^ ions.^39–41^ Cu^+^, which is the major oxidation state of copper within the reducing environment of the cytosol, can readily disproportionate into Cu^2+^ and Cu^0^ in water, thus acting as an effective fluorescent quencher by electron or energy transfer. Cu^2+^ also has quenching capabilities, particularly in aqueous media, because of its redox activity, paramagnetic nature and unfilled d shell.^42^ Due to its unfilled d subshell of Cu^2+^, the transitions of the complex arising after coordination with Cu^2+^ would be largely prohibited d–d transitions with the lowest energy, generating no luminescence. Thus, complex **2** could serve as a switch-off luminescent probe for Cu^2+^ species. A 1 : 2 stoichiometry between complex **2** and Cu^2+^ ions was demonstrated by mass spectrometry, which was consistent with a Job's plot and a Benesi–Hildebrand linear analysis plot, and it was calculated that the relative association constant was 1.68 × 10^11^ M^–2^ based on the emission titration data according to the Benesi–Hildebrand equation. Upon the addition of Cu^2+^ ions to an aqueous solution of complex **2** (10 μM), the luminescence emission intensity of **2** decreased (Fig. 2b). At 2.0 equiv. Cu^2+^, the emission of **2** was almost completely quenched. After calculation, the detection limit of **2** was found as low as 13 nM (0.85 ppb). Significantly, adding Cu^2+^ only could result in a nearly total quenching of luminescence, and the emission intensity of **2** showed nearly no enhancement in the presence of other metal ions (Fig. 2c). The selectivity of this probe is due to the ability of the DPA moiety to selectively recognize Cu^2+^. Interestingly, despite the fact that DPA-based switch-on probes for Zn^2+^ have been reported, complex **2** showed little response to Zn^2+^ ions.

**Fig. 3:**
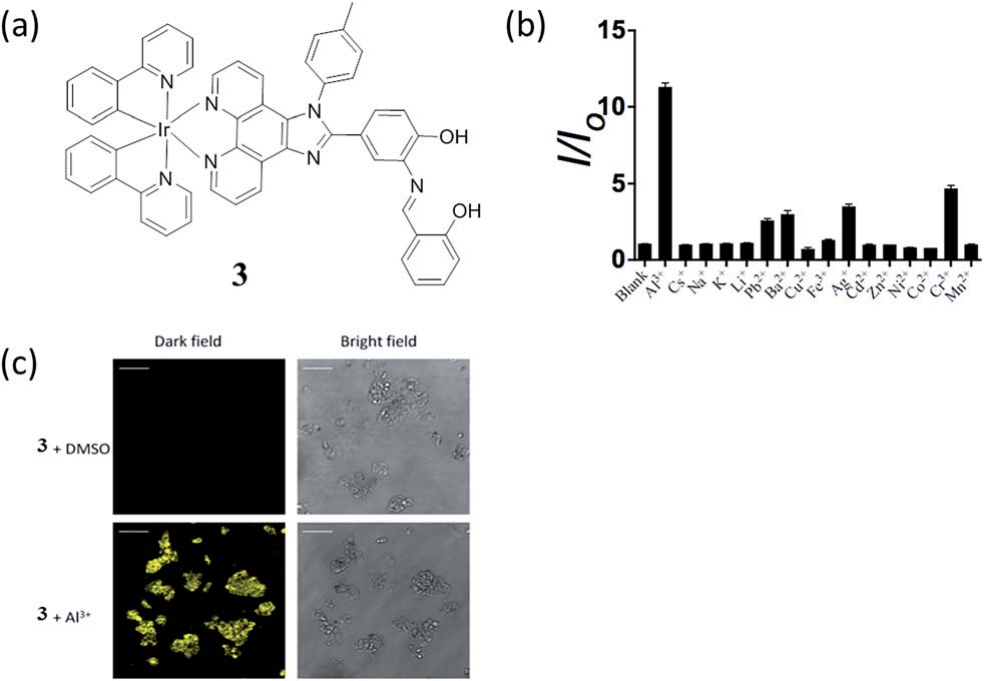
(a) Chemical structure of **3**. (b) Luminescence signal of 20 μM **3** with 25 μM Al^3+^ or 5-fold excess of Cs^+^, Na^+^, K^+^, Li^+^, Pb^2+^, Ba^2+^, Cu^2+^ Fe^3+^, Ag^+^, Cd^2+^, Zn^2+^, Ni^2+^, Co^2+^, Cr^3+^ and Mn^2+^ ions. (c) Luminescence images of HepG2 cells. Cells were pre-incubated with 10 μM of **3** for 1 h, followed by treatment with vehicle control or 100 μM of Al^3+^ for 30 min at 37 °C.^45^ Reprinted figures with permission from Copyright (2016) Royal Society of Chemistry.

Aluminium is one of the most abundant metals on the Earth. Although pure aluminium does not exist naturally, the wide use of aluminium in industry leads to the release of free aluminum ions (Al^3+^) into the environment. In high concentrations, Al^3+^ ions are neurotoxic and can lead to Alzheimer's disease, Parkinson's disease or organ damage.^43,44^ Our group has recently reported an Al^3+^ chemosensor, in which the N^N donor of the iridium(iii) complex **3** incorporates an Al^3+^ binding π-conjugated Schiff base receptor, *o*-phenolsalicylimine (PSI) (Fig. 3a).^45^ The lowest unoccupied molecular orbital (LUMO) of **3** is localized on the PSI moiety.^46–48^ Thus, the binding of Al^3+^ ions at PSI is predicted to strongly affect the LUMO of the iridium(iii) complex so altering the absorption and emission properties of **3**. In acetonitrile, complex **3** displayed a 1–30 μM linear detection range for Al^3+^ ions with a 1 μM detection limit. Additionally, the complex showed selectivity for Al^3+^ ions over other common metal ions, such as Cs^+^, Na^+^, K^+^, Li^+^, Pb^2+^, Ba^2+^, Cu^2+^ Fe^3+^, Ag^+^, Cd^2+^, Zn^2+^, Ni^2+^, Co^2+^, Cr^3+^ and Mn^2+^ (Fig. 3b). This selectivity may be attributed to the small size of Al^3+^ ions (ionic radius of Al^3+^ ion is about 50 pm, whereas most metal ions have radii of >70 pm) as well as its high charge, thus the PSI ligand provides an appropriate hard-base binding motif to recognize Al^3+^, a hard-acid.^49,50^ Indeed, the 62 pm Ga^3+^ ion of gallium, which in the same group as aluminium, also shows triggers a signal response for most Al^3+^ chemosensors,^51^ however, Ga^3+^ was not investigated in this study. The long lifetime luminescence of **3** could be recognized in coumarin (Cm) spiked sample *via* TRES measurement and the bioimaging application of **3** for sensing Al^3+^ ions in living HepG2 cells was also demonstrated (Fig. 3c).

## 3 Cyclometalated iridium(iii) complexes as chemosensors for the detection of anions

**Fig. 4:**
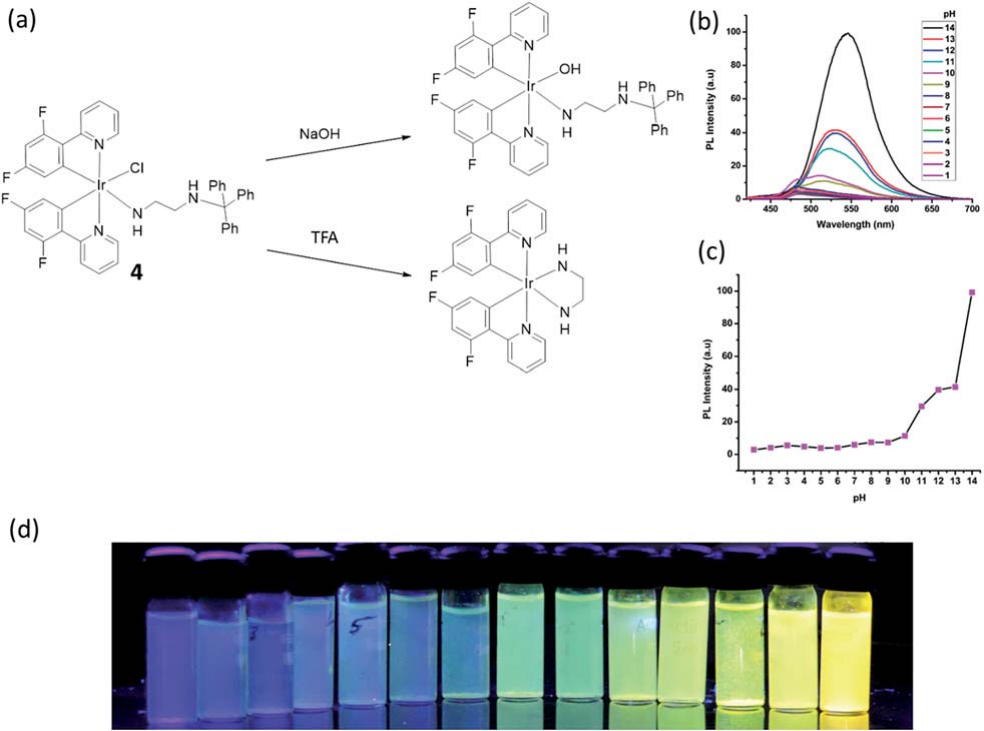
(a) Chemical structure of **4** and reactions with NaOH or TFA. (b) The pH-Dependent luminescent spectra of **4** with in different pH buffer solution (THF/buffer, 1 : 9). (c) The pH-Dependent intensity plot at different pH values. (d) Fluorescent photos of **4** at different pH values at 365 nm.^53^ Reprinted figures with permission from Copyright (2015) American Chemical Society.

pH plays an important role in a host of cellular processes, and can control enzyme activity in various compartments in cells.^52^ Laskar and co-workers developed a greenish-blue emissive bis-cyclometalated iridium(iii) complex **4** containing a *N* ^1^-tritylethane-1,2-diamine ligand which was coordinated to iridium(iii) in a nonchelating fashion (*i.e.* as a monodentate ligand) (Fig. 4a).^53^ Complex **4** displayed intense absorption bands at *ca.* 270–310 nm, moderate bands at 310–400 nm, and a less intense tailing band beyond 400 nm, which was attributed to spin-allowed ^1^IL (π → π*) (N^C), ^1^MLCT (dπ(Ir) → π*(N^C)), and spin-forbidden ^3^MLCT (dπ(Ir) → π*(N^C)) transitions, respectively. In the presence of acid, the trityl group is deprotected and the remaining ethylenediamine moiety is free to chelate to the iridium(iii) center in a bidentate fashion, after displacement of the chloride ligand. On the other hand, the addition of hydroxide ions promotes a simple ligand substitution reaction with a hydroxide ion replacing the chloride ion. Interestingly, both reactions lead to a change in the color of emission of **4**. The original emission intensity of **4** becomes quenched by about 63 times in the presence of trifluoroacetic acid (TFA) (15 μM), while the addition of 1 M NaOH to the solution of **4** shifted the maximum emission by 60 nm, *i.e.*, from 485 to 545 nm, while the emission intensity was dramatically enhanced by about 8 times. Quantitatively, the complex functioned better as a hydroxide ion sensor (in 1 : 9 THF/buffer solution), and the detection limit of hydroxide ion was determined to be 126 nM. However, the pH detection range was only 10–14, which may restrict its practical use (Fig. 4b–d).

**Fig. 5:**
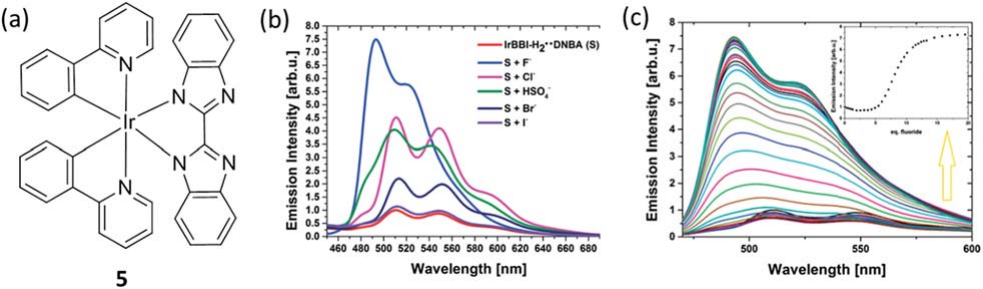
(a) Chemical structure of complex **5**. (b) Luminescence intensity of IrBBI–H_2_···DNBA with 110 equiv. of different anions. (c) Luminescence of IrBBI–H_2_···DNBA as a function of different concentration of fluoride; inset: plot of the normalized IrBBI–H_2_···DNBA luminescence response as a function of the number of equivalents of fluoride added.^54^ Reprinted figures with permission from Copyright (2015) Royal Society of Chemistry.

Numerous efforts have been devoted to the development of methods for the recognition and detection of anions due to their important roles in many chemical and biological processes. Rau and co-workers employed a proton coupled electron transfer quenching mechanism and an iridium(iii) bibenzimidazole (BBI) complex **5** to construct a sensitive detection assay for anions (Fig. 5a).^54^ In the first stage, the 3,5-dinitrobenzoate anion (DNBA) was utilized to quench the luminescent excited state of IrBBI–H_2_, which effectively switches its luminescence “off”. Then, the IrBBI–H_2_^+^···DNBA^–^ system was titrated with five different kinds of anions. Ions that can act as H-bond acceptors from the IrBBI–H_2_–DNBA ion pair and replace the DNBA ion will be able to recover the luminescence of the chromophore (Fig. 5b and c). The results showed that the luminescence response of the IrBBI–H_2_^+^···DNBA^–^ system was increased by the addition of the anions, with enhancements of 750% (fluoride), 450% (chloride), 405% (hydrogen sulfate), 220% (bromide) and 118% (iodide) at 110 equiv. of the anions in chloroform solution (Fig. 5b). Two general mechanisms could lead to the enhancement of luminescence of the complex. Basic anions such as fluoride can deprotonate the acidic secondary amine functionalities of the cationic iridium complex, which leads to a hypsochromic shift and a change in the vibronic structure of the emission spectrum. In the second mechanism, weakly basic anions such as Cl^–^, HSO_4_^–^, Br^–^ and I^–^ establish competing H-bonds to displace DNBA. Rau and co-workers recently reported improved IrBBI–H_2_-based complexes by using a DNBA-free strategy, in which the high acidity of the N–H protons allows the complex to be more easily deprotonated in the presence of strongly basic anions or to form hydrogen bonds with weakly basic anions with distinct emission responses.^55^ The exploration of this interesting type of mechanism, including the pocket effect on anion binding affinity, deserves further attention.

**Fig. 6:**
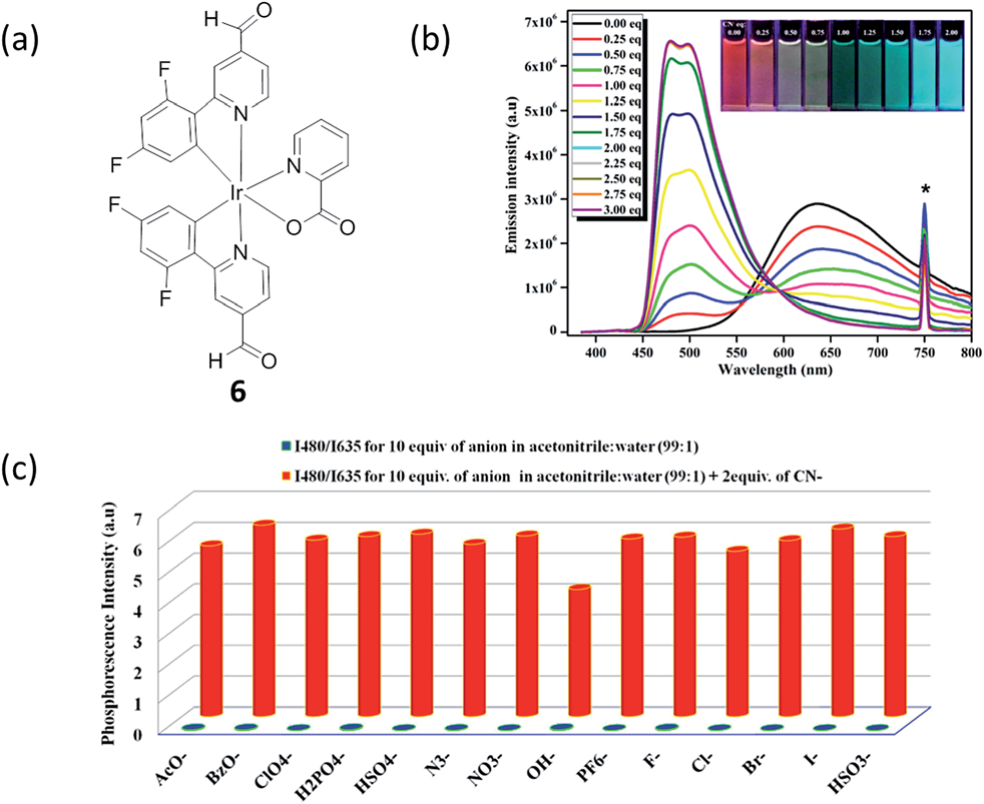
(a) Chemical structure of complex **6**. (b) Photoluminescence titration of **6** (*c* = 20 μM) with CN^–^ solution (0.0–3.0 equiv.) in acetonitrile at 298 K (*λ*_exc_ = 375 nm). Inset: photograph showing the transition of reddish orange to sky blue phosphorescence upon addition of 0.0 to 2.0 equiv. of CN^–^, under 365 nm hand-held UV excitation. At 750 nm the asterisk indicates the second harmonic peak of the excitation wavelength. (c) Phosphorescence response of **6** toward CN^–^ and other anions as measured in 1% aqueous acetonitrile solution (*λ*_exc_ = 375 nm).^57^ Reprinted figures with permission from Copyright (2016) American Chemical Society.

Cyanide is an acutely toxic inorganic anion that poses huge dangers to human health and the environment.^56^ The highest acceptable concentration of cyanide in drinking water is 1.9 μM, as per the requirements of the World Health Organization. Although it is extremely toxic, various compounds containing cyanide are still employed in various industrial processes, such as gold mining, electroplating, and different metallurgical industries, which can result in the leakage of cyanide into the aquatic environment. As a result, there is great interest in the specific detection of soluble cyanide at submicromolar levels. Reddy and co-workers synthesized a phosphorescent iridium(iii) complex, bis[2′,6′-difluorophenyl-4-formylpyridinato-*N*,C4′]iridium(iii) (picolinate) (complex **6**, Fig. 6a), for the detection of CN^–^ on the basis of the widely known cyanohydrin forming reaction.^57^ The room-temperature emission profile of **6** is featureless and broad with a full-width at half-maximum value of about 166 nm, which indicates the dominance of a ^3^MLCT excited-state emission rather than a LC ^3^π–π* excited-state emission. Complex **6** alone in acetonitrile exhibits a rather weak and broad emission profile with an emission maximum at 635 nm. Adding 2.0 equiv. of CN^–^ to an acetonitrile solution of **6** (20 μM) led to a *ca.* 155 nm (0.63 eV) blue shift of the emission maximum (Fig. 4b), changing the emission color changed from red to sky blue in the presence of CN^–^ (inset of Fig. 6b). The emission intensity was greatly enhanced (536-fold at 480 nm) with a quantum yield of 0.11, and a detection limit of 0.02 μM for CN^–^ was reported. Additionally, good selectivity toward cyanide was shown under the optimized conditions, using a ratiometric mode of detection (Fig. 6c).

**Fig. 7:**
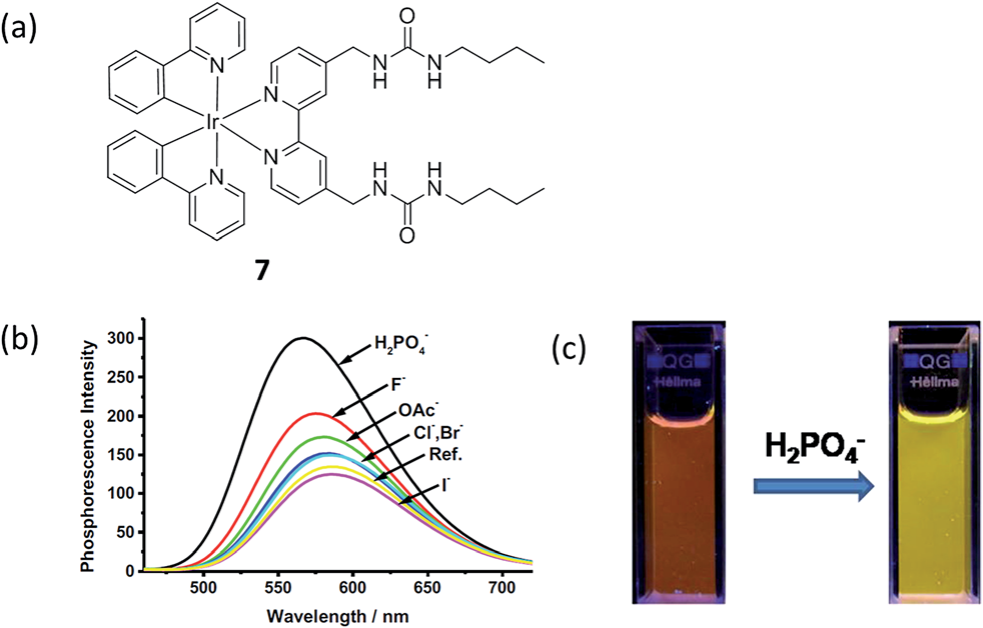
(a) Chemical structure of complex **7**. (b) The phosphorescence spectra of iridium(iii) complex **7** (10 μM) upon addition of different anions (10 eq.) in acetonitrile. (c) Phosphorescence changes of iridium(iii) complex **7** upon the addition of tetrabutylammonium dihydrogenphosphate in acetonitrile. Excitation wavelength: 365 nm.^10^ Reprinted figures with permission from Copyright (2014) Elsevier.

Hyun and co-workers reported a phosphorescence iridium(iii) complex **7** as a chemosensor for H_2_PO_4_^–^ (Fig. 7a).^10^ The complex contains a preorganized binding pocket to selectively recognize H_2_PO_4_^–^. The addition of H_2_PO_4_^–^ (100 equiv.) led to an increase of the phosphorescence of iridium(iii) complex **7** in acetonitrile, with a color change from reddish orange to yellow (560 to 546 nm, 0.06 eV shift) (Fig. 7b), which could be observed with the naked eye (Fig. 7c). From the phosphorescence titration result, the association constant was calculated to be 1.06 × 10^5^ M^–1^. However, the detection limit of complex **7** for H_2_PO_4_^–^ was not reported. The phosphorescence enhancement of **7** with H_2_PO_4_^–^ can be due to the enhancement in the energy level of the triplet excited state which was indicated by the blue shift of the phosphorescence. The intersystem crossing efficiency was enhanced, as well as the triplet quantum yield, which therefore results in a “switch on” luminescent signal. The result of Job's plot assay indicated a 1 : 1 ratio between complex **7** and H_2_PO_4_^–^. However, one limitation of this probe was that it only showed modest selectivity for H_2_PO_4_^–^ over other anions such as fluoride and acetate.

## 4 Cyclometalated iridium(iii) complexes as chemosensors for the detection of small molecules

**Fig. 8:**
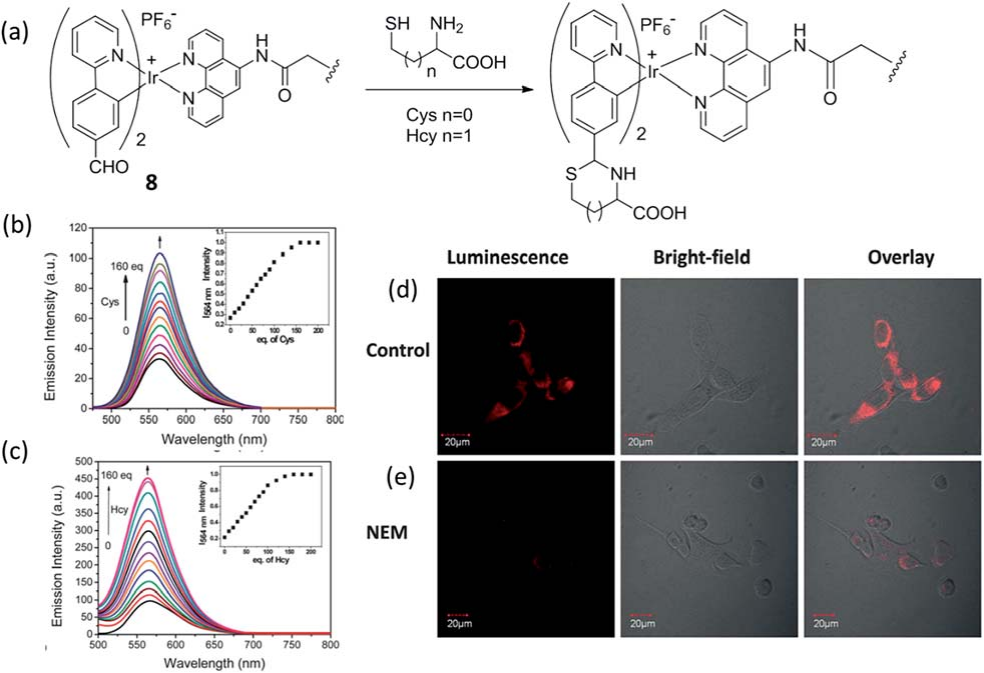
(a) Chemical structure of polymer **8** and its reaction as a dual phosphorescent probe for Cys/Hcy. (b and c) Phosphorescence emission spectra of **8** (20 mM) in PBS at 25 °C with various concentration of Cys or Hcy (0–160 eq.). Inset: titration curve of **8** with Cys or Hcy (0–160 eq.). (d) Phosphorescence images of **8** in KB cells. KB cells incubated with 50 μM **6** for 45 min. (e) KB cells pretreated with 200 μM *N*-ethylmaleimide for 30 min followed by further incubation with 50 μM **8** for 45 min.^61^ Reprinted figures with permission from Copyright (2013) Royal Society of Chemistry.

Cysteine (Cys) and homocysteine (Hcy) are relevant to a wide range of physiological activities, such as the formation and growth of cells and tissues.^58^ However, the abnormal concentration of these biothiols can be indicator of many diseases, such as slowed growth, liver damage, dementia, edema, Alzheimer's disease and Parkinson's disease.^8,59,60^ Huang and co-workers reported a water-soluble phosphorescent polymer by utilizing an iridium(iii) complex as a signaling unit linked with the polymer poly(*N*-isopropylacrylamide) (PNIPAM).^61^ The proposed method was based on the fact that the iridium(iii) complex, which contained an aldehyde groups can form thiazolidine and thiazinane through the reaction with β- and γ-aminothiol groups, respectively, leading to variation of the emission response of the iridium(iii) complex and thus allowing the measurement of Cys and Hcy (Fig. 8a). The polymer **8** had an average molecular weight of 2704, corresponding to about 2 to 3 units of iridium(iii) complex per polymer. **8** shows intense absorption bands below 320 nm, moderately intense bands at 320–400 nm, and weak bands above 400 nm, which are attributed to the spin-allowed ligand-centered transitions (^1^LC), ^1^MLCT and ^3^MLCT, respectively. With Cys/Hcy, the emission response of polymer **8** at 564 nm was increased significantly, which allows for measurement within the visible light region (Fig. 8b and c). Based on time-dependent density functional theory (DFT) calculations, the authors suggested that the increase of emission intensity of **8**-Cys might be due to its different excited-state properties compared to **8**. In addition, due to differences in dipole moment, **8** may be affected more significantly by the aqueous environment compared to **8**-Cys, thus leading to greater dissipation of the excited-state energy through nonradiative mechanisms. The application of **8** for the luminescent imaging of living human nasopharyngeal epidermal carcinoma (KB) cells was also demonstrated using confocal luminescence microscopy. After incubation of cells with 50 μM of **8** in PBS for 45 min at 25 °C, intracellular luminescence was observed obviously (Fig. 8d). However, when pretreated with 200 μM *N*-ethylmaleimide (NEM, as a thiol-reactive compound), KB cells displayed very weak luminescence (Fig. 8e), indicating that polymer **6** interacted with Cys/Hcy in living cells. Importantly, polymer **8** showed little cytotoxicity (*ca.* 98% cell viability at 100 μM of **8**) for 24 h. Moreover, the phosphorescent nature of the polymer was used to demonstrate that the emission response of the polymer to Hcy could be effectively distinguished from the fluorescence of spiked fluorescein by the use of TRES. The selectivity of the polymer for Cys/Hcy was also demonstrated. Finally, the probe could also respond to temperature *via* phase changes in the temperature-sensitive PNIPAM moiety, which releases water molecules associated around the side-chain isopropyl groups when the temperature increases beyond a critical point.

**Fig. 9:**
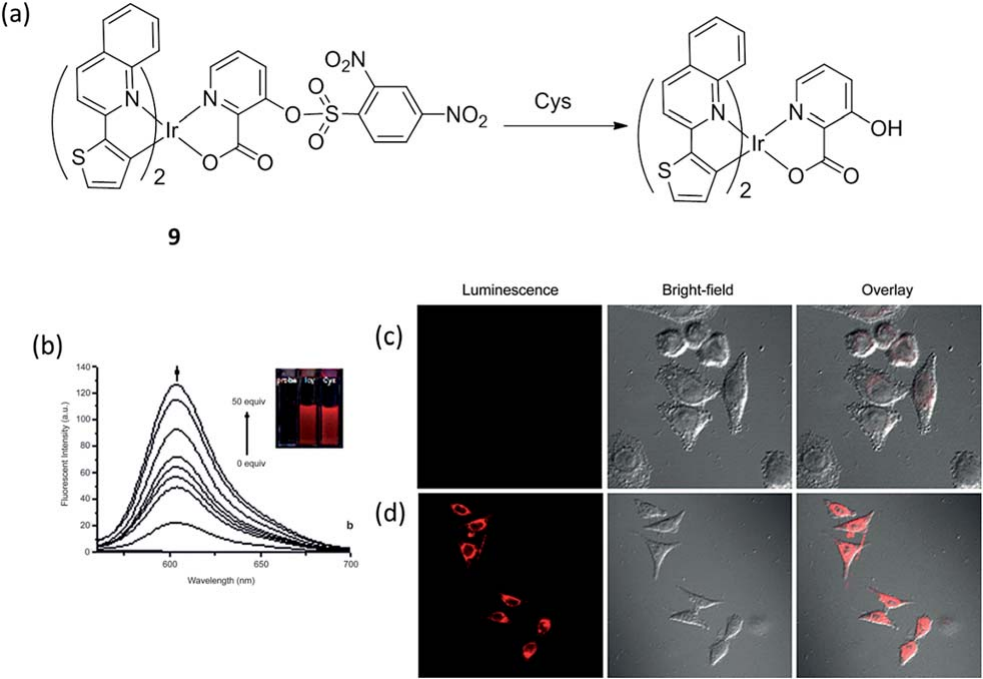
(a) Chemical structure of complex **9** and its reaction with Cys. (b) Changes in fluorescence spectra of **9** (8.14 × 10^–5^ M) in acetonitrile–H_2_O (4 : 1, v/v, pH 7.2) with different amounts of Cys (0–50 equiv.). (c) Luminescence images of **9** in Hela cells. Hela cells incubated with 200 μM *N*-ethylmaleimide for 30 min and then further incubated with **9** (20 μM) for 30 min. (d) Hela cells are incubated with **9** (20 μM) only for 30 min.^12^ Reprinted figures with permission from Copyright (2012) Royal Society of Chemistry.

Huang and co-workers reported another phosphorescent probe using the iridium(iii) complex **9** including a 2,4-dinitrobenzenesulfonyl (DNBS) group on its ligand as the reaction site for biothiols (Fig. 9a).^12^ The DNBS group is a strong electron acceptor, which can lead to complete quenching of probe phosphorescence through a process of electron transfer (ET), thus, complex **9** is initially non-luminescent in acetonitrile–H_2_O. After adding Cys or Hcy, the sulfonate ester of the sensor is cleaved by thiols *via* a nucleophilic aromatic substitution reaction, and the intense-red phosphorescent emission of the complex at 603 nm was restored (Fig. 9a). The emission of **9** increased with the increasing concentration of Cys (Fig. 9b), allowing it to be used for the quantitative determination of Cys concentrations (0–3.26 × 10^–3^ M). Furthermore, the red luminescence of **9** in the presence of Cys can be observed by the naked eye (Fig. 9b, inset). Meanwhile, complex **9** also showed good sensitivity for Hcy, and was selective for biothiols over other amino acids. The ability of **9** to detect Cys/Hcy within living HeLa cells was further demonstrated by confocal luminescence imaging (Fig. 9c and d). Finally, the ability of complex **9** detect Cys/Hcy in the presence of the fluorescent BODIPY was demonstrated by TRES.

**Fig. 10:**
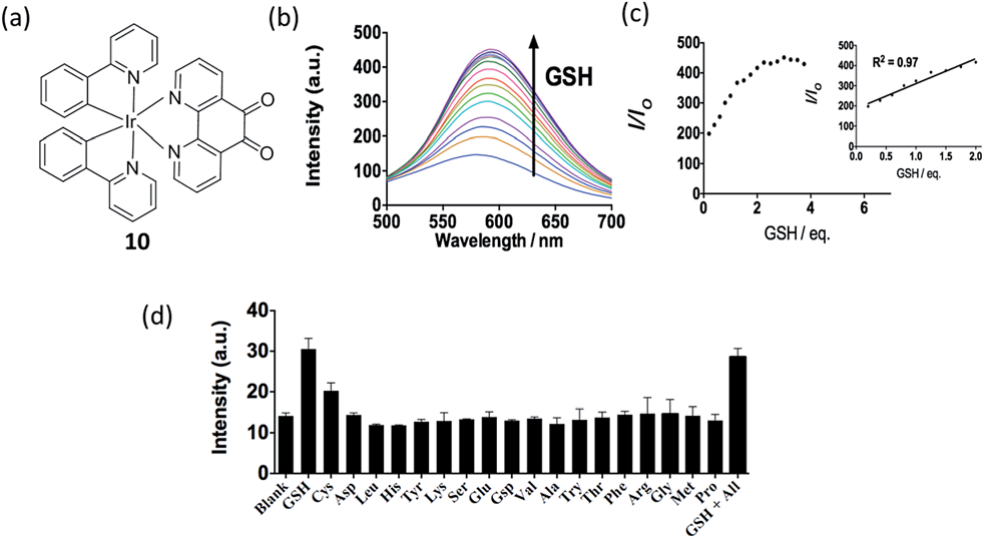
(a) Chemical structure of **10**. (b) Luminescence spectra of **10** (20 μM) with different level of GSH (0–3.75 eq.) in DMSO–HEPES (9 : 1) (10 mM, pH 7.0). (c) The relationship between luminescence intensity and GSH level. (d) Luminescence signal of 20 μM **10** with 0.8 molar equivalents of GSH and Cys or 1.0 molar equivalent of other amino acids in DMSO–HEPES (9 : 1) (10 mM, pH = 7.0).^65^ Reprinted figures with permission from Copyright (2016) Thomson Reuters.

Glutathione (GSH) levels are greatly related to redox homeostasis *in cellulo*.^59,60^ Dysregulation of GSH activity has been associated with different diseases such as cancer, cystic fibrosis and neurodegenerative diseases.^62^ GSH, as a common on-protein thiol, is a main reductant in internal cellular compartments.^63,64^ Our group has reported a iridium(iii) complex **10** containing a 1,10-phenanthroline-5,6-dione (phendione) moiety as the N^N donor (Fig. 10a).^65^ Reducing the phendione N^N donor by GSH is predicted to strongly affect the LUMO of the iridium(iii) complex. This alters the absorption and emission properties of iridium(iii) complex, thus allowing **10** to serve as a luminescent probe for thiol detection. The linear determination range of **10** for GSH in DMSO : HEPES 9 : 1 is between 0.2–2 molar equivalents of GSH, with a detection limit of 1.67 μM (Fig. 10b and c). Complex **10** also displayed good selectivity for thiols over other amino acids (Fig. 10d).

**Fig. 11:**
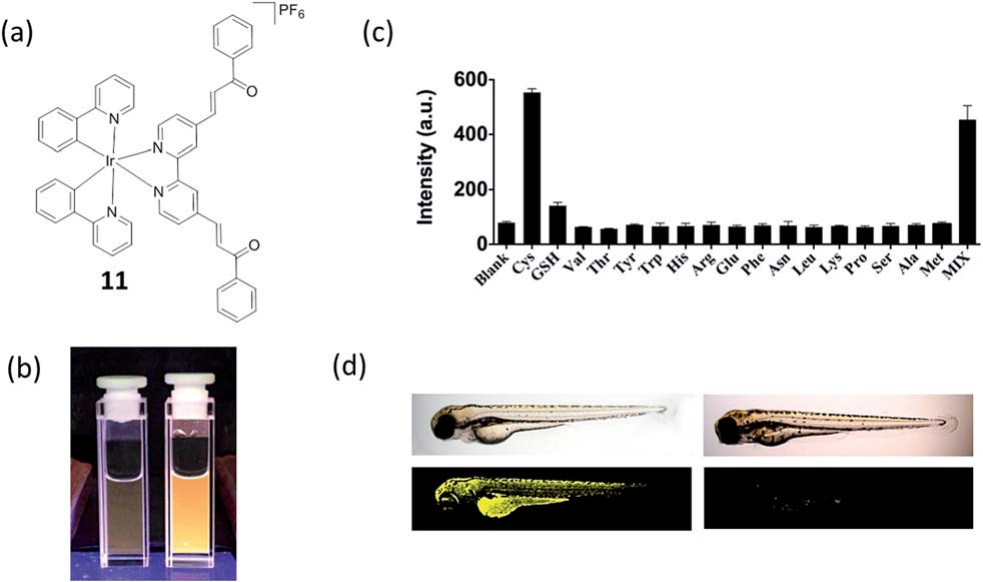
(a) Chemical structure of **11**. (b) Photograph images of **11** (10 μM) in the (left) absence and (right) presence of 80 μM Cys under UV illumination in DMSO–HEPES (10 mM, pH 7.0, 4 : 1 v/v). (c) Luminescence response of 10 μM **11** with Cys or 5-fold excess of common amino acids. (d) *In vivo* images of zebrafish by treatment of **11**. (left) 3 day-old zebrafish was incubated with 10 μM of **11**. (right) 3 day-old zebrafish was pre-incubated with 200 μM NEM for 15 min and then incubated with 10 μM of **11** for 30 min. Upper panels show phase contrast images and lower panels show luminescence images.^26^ Reprinted figures with permission from Copyright (2016) Royal Society of Chemistry.

One of the ultimate goals of chemosensors is investigation of biological process *in vivo*. Our group has also reported an iridium(iii) complex **11** carrying a Cys recognition unit (α,β-unsaturated ketone) that functioned as a Cys chemosensor, and demonstrate its application in live zebrafish (Fig. 11a).^26^ The α,β-unsaturated ketone moiety is a known Cys recognition unit. The binding of Cys will influence the p–π conjugation between the ketone and the pyridine moiety, thus allowing the complex to be used as a luminescent probe for Cys.^66^ The measured detection range was 2.5–80 μM with a detection limit of 0.78 μM (Fig. 11b and c). The microsecond lifetime phosphorescence of complex **11** could be determined in biological samples with high autofluorescence *via* TRES. Additionally, we showed that the complex could be used for the detecting and imaging of Cys in living zebrafish (Fig. 11d). This study further demonstrates the potential application of phosphorescent complexes in living tissues. Overall, we consider that complex **11** may be the most suitable biothiol chemosensor out of the examples highlighted in this review, due to its relatively simple structure, high sensitivity and demonstrated capability to detect Cys in live zebrafish.

**Fig. 12:**
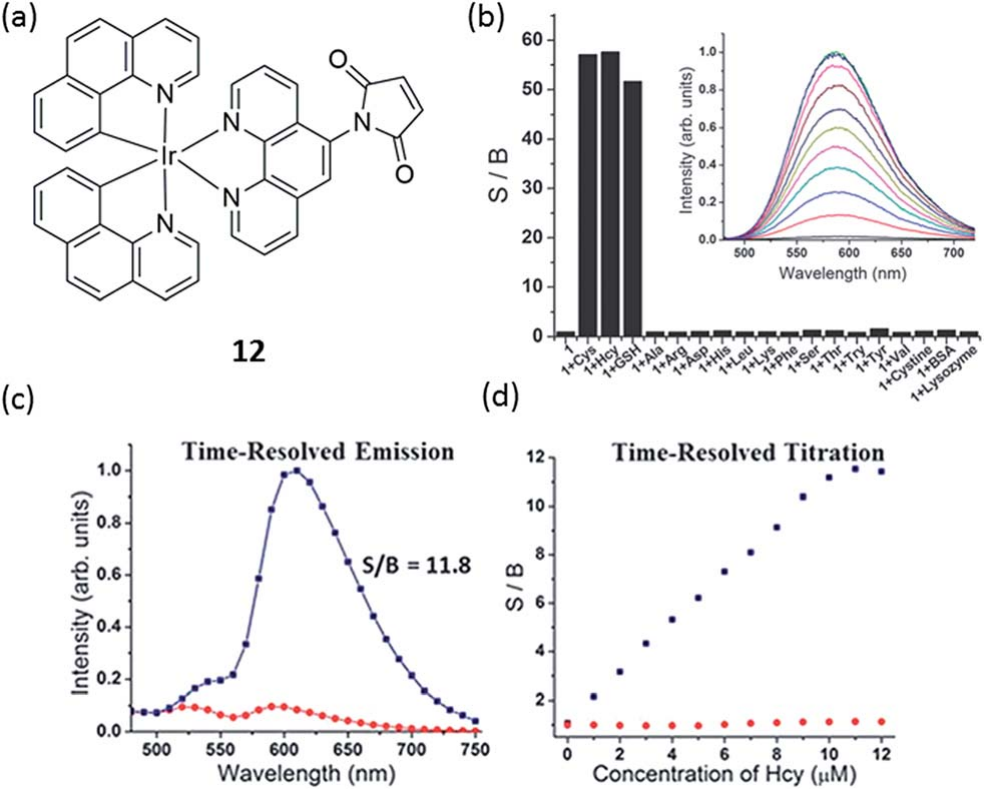
(a) Chemical structure of **12**. (b) Relative photoluminescence intensity of 10 mM probe **12** at 590 nm before and after addition of 10 mM of various amino acids, GSH, cysteine and proteins (PBS 6.7 mM, pH 7.2, 1% DMF; incubation at room temperature for 1 min after mixing). S/B ratio is the ratio of the probe photoluminescence after and before the addition of the amino acid. Inset: photoluminescence spectra of **12** with different concentrations of Cys. (c) Time-resolved emission of a 10 mM solution of **12** in Dulbecco's Modified Eagle medium (1% DMF) with (blue line) and without (red line) addition of 10 mM cysteine. (d) Titration of cysteine to a 10 mM solution of **12** in Dulbecco's Modified Eagle medium (1% DMF) using a steady-state fluorometer (red points) and a time-resolved fluorometer with time-gating from 60 to 150 ns (blue points).^67^ Reprinted figures with permission from Copyright (2012) Royal Society of Chemistry.

Another example for the detection of thiol containing amino acids has been reported by Martí and co-workers, in which an iridium(iii) complex **12** containing 1,10-phenanthroline-5-maleimide ligand was synthesized (Fig. 12a).^67^ Complex **12** displays very little photoluminescence in aqueous solution, while it exhibits up to a 60-fold increase in photoluminescence in the presence of small thiol-containing amino acids such as Cys, Hcy and GSH with high selectivity (Fig. 12b). It is important to note that the reaction of **12** with lysozyme (8 Cys) and bovine serum albumin (BSA) (35 Cys) did not cause any increase in photoluminescence. This can be explained by the fact that all Cys in lysozyme and 34 out of 35 Cys in BSA are participating in disulfide bonds, and are thus unable to react with **12**.^68^

Based on DFT calculation of the frontier orbitals of **12** and its adduct with Cys, the LUMO of **12** is located at the maleimide group while the LUMO of **12**-Cys is phen(π*). Given that **12**-Cys is photoluminescent, it can be concluded that the population of this phen(π*) excited state results in a radiative deactivation to the ground state. The lack of photoluminescence from **12** is consistent with the population of the maleimide molecular orbitals in the excited state, which is lower in energy than the LUMO of **12**-Cys, and which decays to the ground state by non-radiative pathways. Furthermore, **12** has the additional advantage of having a relatively long photoluminescence lifetime (for **12**-Cys the average lifetime is 99.2 ns), which could be used to detect thiol-containing amino acids in media with high autofluorescence background by using time-resolved methods (Fig. 12c), thus extending its potential applications in clinical diagnostics.

**Fig. 13:**
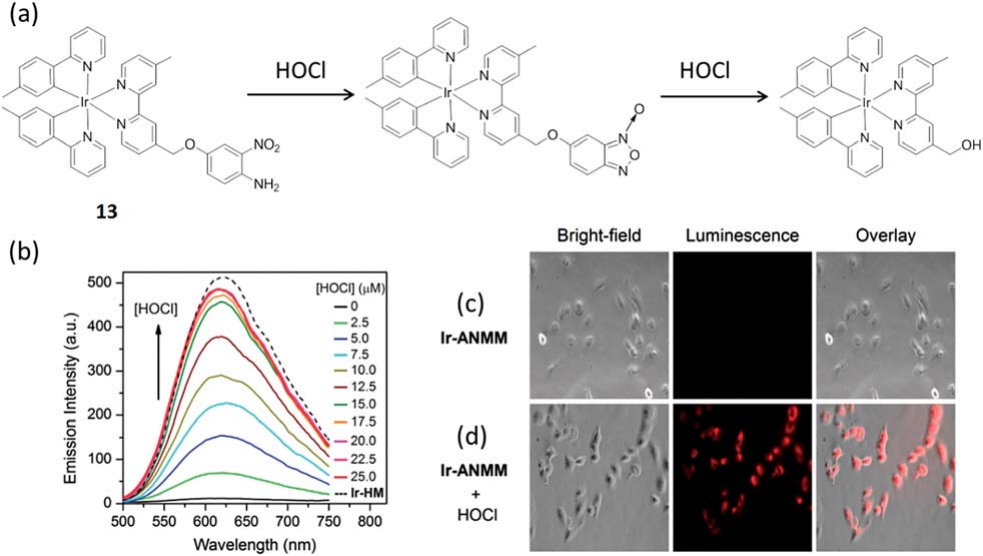
(a) Chemical structure of complex **13** and its reaction with HOC. (b) The luminescent spectral changes of complex **13** (5 μM) upon increasing addition of HOCl (from 0 to 25 μM) in acetonitrile–0.1 M phosphate buffer (1 : 4, v/v, pH 7.4). *λ*_ex_ = 400 nm. (c) Images of the cells incubated with **13** (20 μM) only. (d) Images of complex **13**-treated cells after treatment with HOCl (50 μM).^70^ Reprinted figures with permission from Copyright (2014) Royal Society of Chemistry.

Hypochlorous acid (HOCl) is a reactive oxygen species which is widely used in daily life as a bactericide and a bleaching agent. Being a common byproduct of cellular metabolism, endogenous HOCl is important in several biological activities.^69^ However, hypochlorite solutions with high concentration can be potentially harmful to health due to the possibility to cause tissue damage and disease by excessive HOCl. Nabeshima and co-workers synthesized an iridium(iii) complex **13** substituted with an *o*-nitroanilino group as a chemodosimeter for HOCl (Fig. 13a).^70^ In the absence of HOCl, complex **13** possesses very weak emission in 1 : 4 acetonitrile–0.1 M phosphate buffer due to PET by the electron-rich *o*-nitroanilino group. However, in the presence of HOCl, the *o*-nitroanilino group can be oxidized, which facilitates cleavage of the 4-amino-3-nitrophenyloxy moiety. This generates a hydroxymethyl-appended complex, which exhibits strong luminescence due to the absence of the PET effect. Upon treatment with an increasing level of HOCl, the emission signal was increased and reached a maximum when 4 equiv. of HOCl was introduced. According to the linear equation, the detection limit was 16 nM at a signal to noise ratio (S/N) of 3. Meanwhile, the feasibility of complex **13** for imaging HOCl in Hela cells was demonstrated.

**Fig. 14:**
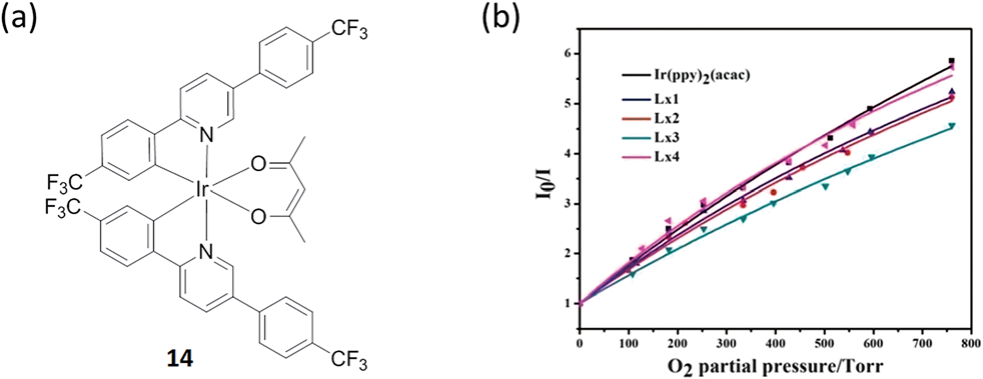
(a) Chemical structure of complex **14**. (b) Stern–Volmer plots for oxygen sensing films of the iridium(iii) complexes immobilized in ethyl cellulose. Intensity ratios *I*_0_/*I versus* O_2_ partial pressure.^71^ Reprinted figures with permission from Copyright (2015) Royal Society of Chemistry.

Oxygen is involved in practically all forms of living organisms. An iridium(iii) complex **14** (Fig. 14a) with an acetylacetone ligand has been reported as a switch-off oxygen sensor by Liu and co-workers.^71^ The electronic transitions responsible for luminescence in iridium(iii) complexes have been assigned to a mixture of MLCT and ^3^(π–π*) ligand states.^72,73^ These states are composed principally of C^N ligand orbitals, with the β-diketonate ligand acting as an ancillary ligand. Quantum efficiencies and triplet lifetimes are severely reduced by oxygen as singlet oxygen formation is a possible quenching process,^74^ and thus triplet oxygen quenches luminescence at near diffusion controlled rates.^75^ The complex showed intense phosphorescence emission at room temperature with an emission maximum in a range of 523–554 nm, but the emission was substantially quenched by the presence of oxygen. The incorporation of the complex into oxygen-sensitive films was also studied, and showed linear Stern–Volmer behavior over an oxygen concentration range from 0 to 100% (Fig. 14b). It should be noted that many iridium(iii)-based oxygen sensors have been recently developed due to the favorable properties of iridium(iii) complexes,^76–83^ while trifluoromethyl-substituted cyclometalated iridium(iii) complexes for oxygen sensing have not been greatly investigated. The electron-withdrawing character of the trifluoromethyl-substituents increases the redox potentials of the complex and makes the molecules less reactive toward photooxidation processes, thus increasing photostability.

**Fig. 15:**
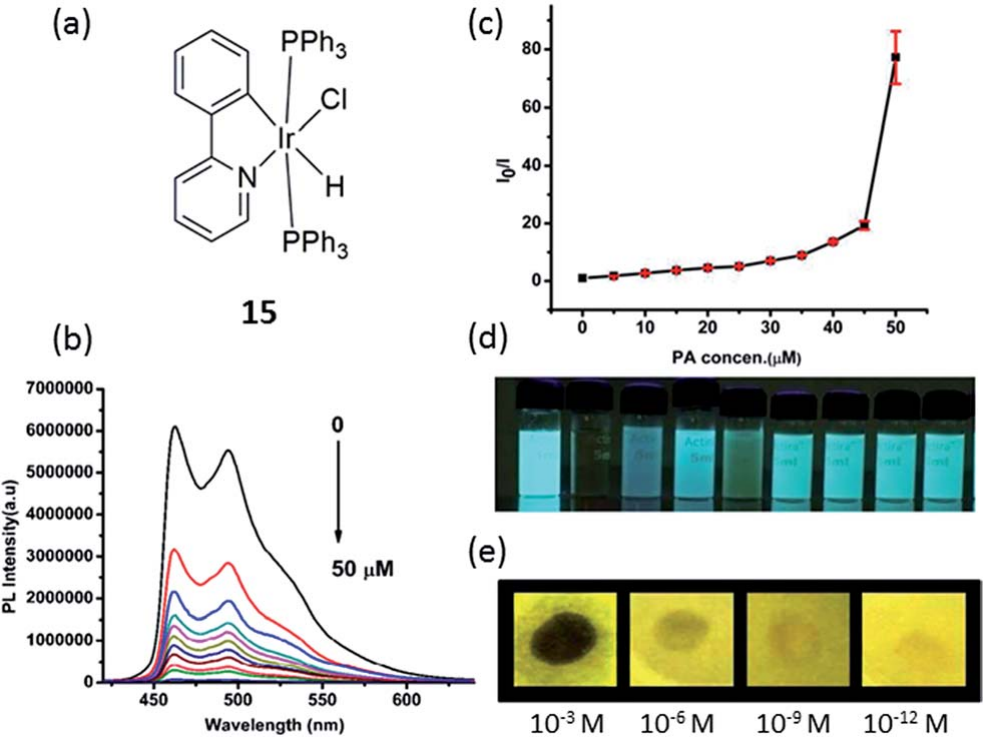
(a) Chemical structure of complex **15**. (b) Photoluminescence spectra of **15** in THF–water (v/v = 1 : 9) with different amounts of PA. (c) Corresponding Stern–Volmer plots of PA. (d) Image of **15** (10 μM) when dispersed in THF–water (v/v = 1 : 9) with addition of 5 equivalents of each explosive/non explosive compounds, respectively (from left to right: blank, PA, 3,5-dinitrotoluene, nitrotoluene, 2,4-DNP, 1,3-dinitrobenzene, nitrobenzene, toluene and benzoic acid). (e) Luminescent photographs of paper plates impregnated by **15** against different concentrations of PA.^84^ Reprinted figures with permission from Copyright (2015) Royal Society of Chemistry.

Nitroaromatics such as trinitrotoluene (TNT), 2,4-dinitrophenol (2,4-DNP) and picric acid (PA) are well-known explosive materials, and a convenient way to detect such compounds is important for security. Laskar and co-workers developed an aggregation-induced phosphorescent emission active iridium(iii) complex **15** for the detection of PA, which in addition to its use a an explosive similar to TNT, is also known as a notorious environmental polluting agent (Fig. 15a).^84^ The experimental observations suggested that both electron and energy transfer quenching mechanisms are responsible for the selective detection of PA by **15**. On the other hand, **15** showed good ability to detect PA with a detection limit of 65 nM (∼99% quenching observed with 5 equivalents PA) and the quenching constant was calculated to be 1.90 × 10^–5^ M^–1^ (Fig. 15b–d). Additionally, a portable detection method for PA was developed by using a filter paper soaked with the complex solutions in dichloromethane.

## 5 Conclusion and outlook

This review has highlighted recent examples of cyclometalated iridium(iii) chemosensors that have been reported in the literature over the past several years. As can be seen from the examples in this review, a variety of mechanisms have been employed for both the switch-on or switch-off detection of analytes, while some studies have been able to exploit a ratiometric mode of detection. Other cation sensors described in this review have mostly relied on the attachment of a specific cation recognition group, such as DPA for Cu^2+^,^36^ and PSI for Al^3+^.^45^ The interaction of the cation with the recognition group either led to an increase or decrease of the phosphorescence of the conjugated iridium(iii) complex.

For anion sensing, a common strategy is to exploit the ability of the anion to act as a base or a hydrogen bond acceptor. However, achieving selectivity between different anions appears to be relatively difficult using this approach, as the phosphorescent response of the complexes were observed to vary by less than an order of magnitude for different anions. This could be due to the fact that the basicity or hydrogen bond accepting capability of the various anions are relatively similar. An exception to this is the reaction-based chemosensor for cyanide ions reported by Reddy and co-workers, which relied on the specific cyanohydrin reaction between an aldehyde group attached to the C^N ligand and the target ion in order to generate selectivity.^57^ The complex therefore showed very little response even to 10-fold excess of unrelated anions, and additionally, could offer a ratiometric mode of detection due to the change in emission wavelength of the complex upon cyanohydrin formation. In general, ratiometric or switch-on modes of detection are preferable to a switch-off detection method as these are less susceptible to false positives arising due to non-specific quenching species in the sample matrix.

The examples highlighted in this review also highlight a major advantage of using phosphorescent metal complexes as chemosensors, which is that their long-lived phosphorescence (Table 1) can be distinguished from a fluorescence background by the use of TRES. This has been demonstrated by a phosphorescent Al^3+^ chemosensor,^45^ as well as by chemosensors for biothiols.^26^ This is highly pertinent when dealing with biological samples, and to a lesser extent environmental samples, due to the frequent presence of endogenous fluorophores in sample matrices. The use of TRES thus allows the phosphorescent response of the complex to the target analyte to be efficiently detected even in the presence of background fluorescence.

**Table 1:** The quantum yields and lifetimes of complexes **1–15**

Complex number	Quantum yield (QY)	Lifetime
**1**	0.067% (solution), 7.3% (solid) (reference QY = 0.55)	19.30 ns (solution), 2.00 μs (solid)
**2**	30.04%	10.85 μs
**3**	0.0082 (reference QY = 0.067)	4.20 μs
**4**	3.08% (reference QY = 0.546)	1.30 μs
**5**	Not given	Not given
**6**	0.02 or 0.11 (in the absence or presence of analyte)	0.06 μs or 0.75 μs (in the absence or presence of analyte)
**7**	Not given	0.34 μs or 0.60 μs (in the absence or presence of analyte)
**8**	0.023 (in air-equilibrated PBS, reference QY = 0.028)	366 ns (in air-equilibrated PBS)
**9**	Not given	191.7 ns (in an air-equilibrated solution), 3.01 μs (in cellular samples)
**10**	Not available	4.26 μs
**11**	0.0053 (reference QY = 0.067)	4.29 μs
**12**	10.55% (average value, in the presence of analyte, reference QY = 0.028)	99.2 ns (average value, in the presence of analyte)
**13**	0.02 or 0.17 (in the absence or presence of analyte, under anaerobic conditions)	Not given
**14**	0.48 (in degassed dichloromethane, reference QY = 0.34)	1.53 μs (in degassed dichloromethane)
**15**	0.04% (solution), 22.60% (solid)	2.50 ns (solution), 4.20 μs (solid)

However, one limitation of most phosphorescent chemosensors is that they require an organic solvent to function. Most of the iridium(iii) chemosensors highlighted in this review have been tested in pure organic solvent such as acetonitrile or chloroform, or in mixed solvent systems such as 1 : 9 THF–buffer or 1 : 4 acetonitrile–buffer. This may restrict the utility of the chemosensors since most biological or environmental samples are aqueous. Additionally, the fact that none of the examples described here have employed amplification mechanisms means that there is an inherent limit to the sensitivity that can be achieved with these chemosensors. On the other hand, the simplicity of the chemosensors may be construed as an advantage, as the incorporating of additional signal transducing elements necessarily increases the complexity of the assay, which could increase the time and cost required to optimize and also to perform the assay, as well as possibly decrease the robustness of the system.

It is encouraging to see that many of the recent examples have investigated the utility of complexes for sensing analytes in living cells,^12,45,61,70^ or even living organisms such as zebrafish.^26^ This is an important step forward because many of the cations, anions or small molecules that are assayed represent biomarkers or even causative agents of human disease. In this context, the general stability of iridium(iii) metal complexes is suited for their roles as sensors in biological media.^3,7^ A phosphorescent iridium(iii) complex had previously been used to detect hypoxia mouse tumor model, and is now even commercially available.^85^ For biological application, the complexes should preferably show only limited toxicity to mammalian cells. Thus, researchers should also consider cytotoxicity as a factor in the design of phosphorescent chemosensors. Combined with time-resolved measurement or imaging, phosphorescent metal complexes may be able to play key roles in addressing questions about the roles of specific analytes in live biological tissues.

Moving forward, the future of phosphorescent chemosensors appears to be bright. Future designs should focus on the sensitivity and selectivity of the complex for the target analyte, particularly when interfering substances are present. We encourage researchers to demonstrate that the utility of their chemosensors in model systems such as (diluted) cellular extracts or serum, or aquatic environmental samples. Additionally, biocompatibility and aqueous compatibility are secondary objectives that could also enhance the practical application of the chemosensors.
